# Host suppression of the novel virulent effector FocSP1 improves banana resistance to *Fusarium oxysporum* f. sp. *cubense* tropical race 4

**DOI:** 10.1093/hr/uhag008

**Published:** 2026-01-07

**Authors:** Huoqing Huang, Siwen Liu, Yong Zhang, Yushan Liu, Chunhua Hu, Qiong Wang, Heqiang Huo, Mudassar Ahmad, Guiming Deng, Weiqing Zeng, Ganjun Yi, Chunyu Li

**Affiliations:** Institute of Fruit Tree Research, Guangdong Academy of Agricultural Sciences; Key Laboratory of South Subtropical Fruit Biology and Genetic Resource Utilization, Ministry of Agriculture and Rural Affairs; Guangdong Provincial Key Laboratory of Science and Technology Research on Fruit Tree, Guangzhou 510640, China; Institute of Fruit Tree Research, Guangdong Academy of Agricultural Sciences; Key Laboratory of South Subtropical Fruit Biology and Genetic Resource Utilization, Ministry of Agriculture and Rural Affairs; Guangdong Provincial Key Laboratory of Science and Technology Research on Fruit Tree, Guangzhou 510640, China; Department of Biology, Georgia Southern University, Statesboro, GA 30458, USA; Institute of Fruit Tree Research, Guangdong Academy of Agricultural Sciences; Key Laboratory of South Subtropical Fruit Biology and Genetic Resource Utilization, Ministry of Agriculture and Rural Affairs; Guangdong Provincial Key Laboratory of Science and Technology Research on Fruit Tree, Guangzhou 510640, China; Institute of Fruit Tree Research, Guangdong Academy of Agricultural Sciences; Key Laboratory of South Subtropical Fruit Biology and Genetic Resource Utilization, Ministry of Agriculture and Rural Affairs; Guangdong Provincial Key Laboratory of Science and Technology Research on Fruit Tree, Guangzhou 510640, China; Health & Biosciences, International Flavors & Fragrances, Wilmington, DE 19803, USA; Mid-Florida Research and Education Center, Department of Environmental Horticulture, University of Florida, Apopka, FL 32703, USA; Institute of Fruit Tree Research, Guangdong Academy of Agricultural Sciences; Key Laboratory of South Subtropical Fruit Biology and Genetic Resource Utilization, Ministry of Agriculture and Rural Affairs; Guangdong Provincial Key Laboratory of Science and Technology Research on Fruit Tree, Guangzhou 510640, China; Institute of Fruit Tree Research, Guangdong Academy of Agricultural Sciences; Key Laboratory of South Subtropical Fruit Biology and Genetic Resource Utilization, Ministry of Agriculture and Rural Affairs; Guangdong Provincial Key Laboratory of Science and Technology Research on Fruit Tree, Guangzhou 510640, China; Health & Biosciences, International Flavors & Fragrances, Wilmington, DE 19803, USA; Institute of Fruit Tree Research, Guangdong Academy of Agricultural Sciences; Key Laboratory of South Subtropical Fruit Biology and Genetic Resource Utilization, Ministry of Agriculture and Rural Affairs; Guangdong Provincial Key Laboratory of Science and Technology Research on Fruit Tree, Guangzhou 510640, China; Institute of Fruit Tree Research, Guangdong Academy of Agricultural Sciences; Key Laboratory of South Subtropical Fruit Biology and Genetic Resource Utilization, Ministry of Agriculture and Rural Affairs; Guangdong Provincial Key Laboratory of Science and Technology Research on Fruit Tree, Guangzhou 510640, China

Dear Editor,

Banana (*Musa* spp.), a widely cultivated crop in tropical or subtropical regions, is under severe threat from *Fusarium oxysporum* f. sp. *cubense* tropical race 4 (*Foc* TR4), the causative agent of fusarium wilt. This fungus can survive for over 40 years in the soil in the absence of a host [1], yet remains highly infectious upon contact with banana roots. Once inside the plant, *Foc* TR4 colonizes and obstructs the vascular bundles, leading to leaf wilting and eventual plant death. During infection, *Foc* TR4 secretes many effectors that help suppress host immunity and facilitate pathogen infection. Known effectors, such as the SIX (Secreted in Xylem) homologs, FocSIX8, have been shown to be essential for *Foc* TR4 full virulence [2]. However, a critical gap in our knowledge of novel and potent effectors of *Foc* TR4 needs to be filled.

To address this knowledge gap, we employed bioinformatics and RNA-seq approaches [3] and identified a novel effector from *Foc* TR4 with a predicted secreted signal peptide (Fig. 1A), designated *Foc*-secreted protein 1, FocSP1. RT–qPCR analysis revealed that *FocSP1* transcript levels were significantly induced after infection (Fig. 1B). To further validate its expression, we constructed *FocSP1*:*GFP*-expressing strains under the control of either its native promoter or a constitutive *RP27* promoter. Notably, the strain expressing *FocSP1*:*GFP* driven by its native promoter showed no detectable GFP signal when grown on potato dextrose agar (PDA) plates, whereas a strong GFP signal was observed in the *RP27* promoter strain (Fig. 1C). However, both strains showed strong GFP signals during banana root infection (Fig. 1C), indicating that the expression of *FocSP1* was significantly induced in roots regulated by host-derived signals during pathogenesis. Pathogenicity assays showed that the Δ*FocSP1* mutant caused only mild necrosis symptoms in the corm, whereas the wild-type (WT) and the complemented strain Δ*FocSP1*-C induced significantly more severe disease symptoms (Fig. 1D and E). Collectively, these results indicated that *FocSP1* is required for full virulence of *Foc* TR4.

**Figure 1:**
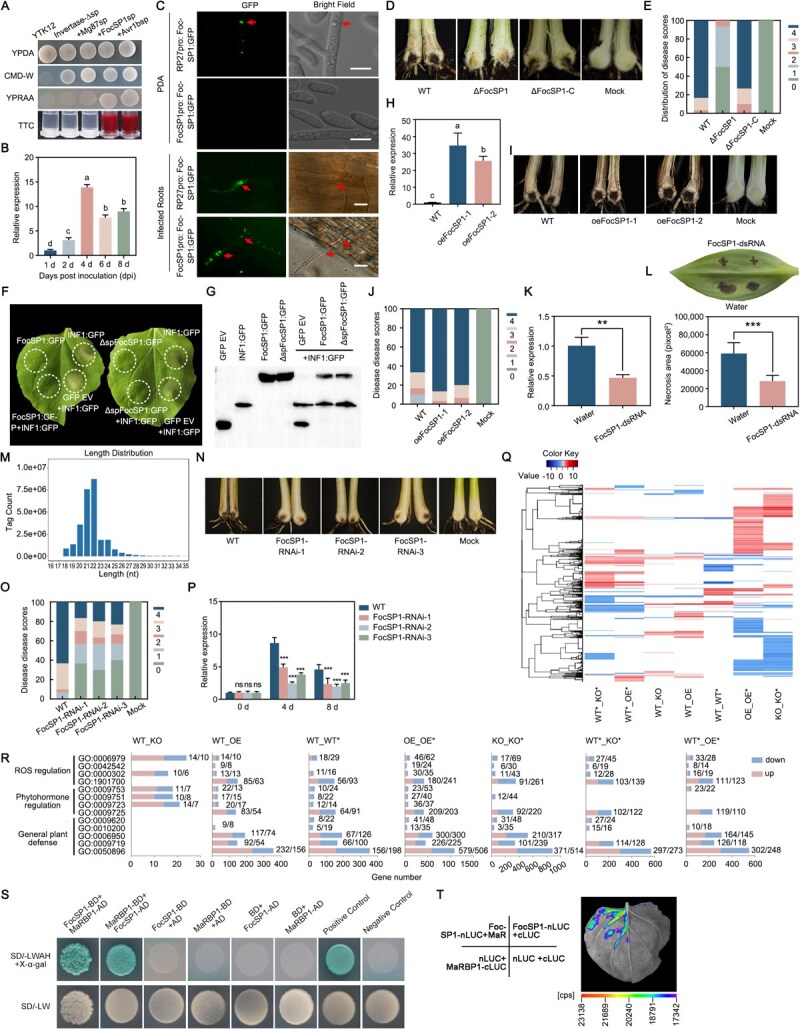
The role of *FocSP1* in *Foc* TR4 pathogenicity and banana immunity. (A) A yeast secretion assay to evaluate the secretion functionality of the FocSP1 signal peptide. Yeast YTK12 strain transformed with either the Invertase-∆sp (pSUC2 empty vector containing an invertase without signal peptide, negative control), Mg87sp (supplemented with non-functional Mg87 signal peptide, negative control), FocSP1sp (supplemented with FocSP1 signal peptide), and Avr1bsp (supplemented with Avr1b signal peptide, positive control) were evaluated for growth on CMD-W or YPRAA medium and for invertase activity assay with 2,3,5-triphenyltetrazolium chloride. (B) RT–qPCR analysis of *FocSP1* expression during the early infection stages of banana roots inoculated with *Foc* TR4. Expression levels were normalized to the fungal reference gene *FocEF1α*. (C) Fluorescence observation of *Foc* TR4 strain carrying the *FocSP1*:*GFP* construct. GFP signals during vegetative growth of *Foc* TR4 (top) were observed in mycelia and conidia after 6 days of culturing on PDA plates, while GFP signals in infected banana roots (bottom) were detected at 4 dpi. Scale bar = 20 μm. (D) Disease internal phenotypes and (E) distribution of plant disease scores in banana plants inoculated with *Foc* TR4, Δ*FocSP1*, and Δ*FocSP1*-C strains for 5 weeks. (F) *Nicotiana benthamiana* leaves 3 days after infiltration with *Agrobacterium tumefaciens* carrying either a *GFP* empty vector (EV) only, *FocSP1*:*GFP*, or Δ*spFocSP1*:*GFP* (*FocSP1* devoid of its signal peptide). For co-infiltration assays, the same leaf areas were subsequently infiltrated with *A*. *tumefaciens* harboring *INF1*:*GFP*. Images were captured 3 days after co-infiltration. (G) Western blotting detection of protein expression in *N*. *benthamiana* leaves using anti-GFP antibody. (H) RT–qPCR analysis of *FocSP1* expression in oeFocSP1 transgenic banana plants and WT plants. (I) Disease internal symptoms and (J) distribution of plant disease scores observed in banana 35 days post-inoculation with *Foc* TR4. (K) Relative expression levels of *FocSP1* in *Foc* TR4 mycelium-infected banana leaves subjected to treatments with *FocSP1*-dsRNA and water by using RT–qPCR. (L) *Foc* TR4-induced necrosis and necrosis area of banana leaves 7 days post-inoculation. The necrosis area was calculated using ImageJ software. (M) Length distribution and abundance of *FocSP1*-specific siRNAs. (N) Disease symptoms and (O) distribution of disease scores observed in banana plants 35 days post-inoculation with *Foc* TR4. (P) RT–qPCR analysis of *FocSP1* gene in roots of FocSP1-RNAi and WT plants at intervals of 0, 4 and 8 days post-inoculation. (Q) Hierarchy clustering of DEGs across seven different pairwise comparisons among WT, oeFocSP1 (OE), and FocSP1-RNAi (KO) with (*) and without *Foc* TR4 II5 infection. The asterisk (*) denotes banana plants infected with *Foc* TR4. Rows in the heat map represent genes and columns indicate different pairwise comparisons. The heatmap displays log₂ fold-change values. According to the scale bar, positive values (represented by darker shading) indicate up-regulated genes, while negative values (represented by lighter shading) indicate down-regulated genes. (R) GO functional annotation of identified DEGs from seven pairwise comparisons. The numbers to the right of the columns represent the count of up-regulated (left) and down-regulated (right) genes, respectively. (S) Yeast two-hybrid (Y2H) assay showing that FocSP1 interacts with MaRBP1. Cotransformants of p53 + pGADT7-T and lam + pGADT7-T were used as positive control and negative control, respectively. SD/-LWAH and SD/-LW represent SD/−leucine-tryptophan–adenine–histidine and SD/−leucine–tryptophan selective medium, respectively. (T) Luciferase complementation imaging assays showing the interactions between FocSP1 and MaRBP1. Tobacco leaves were divided into four parts and infiltrated with *Agrobacterium* strains harboring *FocSP1*-*nLUC* and *MaRBP1*-*cLUC*. The following three pairs of constructs were used as negative controls: *FocSP1*-*nLUC* + *cLUC*, *nLUC* + *MaRBP1*-*cLUC* and *nLUC* + *cLUC*. The images were captured at 3 days post-infiltration. Quantitative data are presented as means ± SDs (*n* = 3). Significance analysis in (B) and (H) was performed using one-way ANOVA through Tukey’s test. Significance analysis in (J) and (O) was performed using the Kruskal–Wallis test. Significance analysis in (K), (L), and (P) was performed using Student’s *t*-test (^**^P < 0.01; ^***^P < 0.001; ns, not significant).

To investigate the mechanistic role of FocSP1 in plant immunity, FocSP1 and INF1 were transiently expressed in *Nicotiana benthamiana* leaves. A GFP-only vector and the *Phytophthora infestans* cell death elicitor INF1 [4] were used as negative and positive controls, respectively. As expected, apparent cell death in *N*. *benthamiana* leaves was induced by INF1 but not FocSP1 (Fig. 1F). Interestingly, co-expression of FocSP1 with INF1 suppressed the cell death typically caused by INF1, while co-expression with the GFP EV had no effect (Fig. 1F). Furthermore, we also found that ΔspFocSP1 (FocSP1 without signal peptide) had the same function as FocSP1 in *N*. *benthamiana* leaves (Fig. 1F). Immunoblotting analysis confirmed equivalent protein accumulation in *N*. *benthamiana* leaves (Fig. 1G). Taken together, these data suggested that FocSP1 inhibits plant immune responses to facilitate *Foc* TR4 infection.

To further investigate the biological function of FocSP1, we generated two transgenic banana lines (oeFocSP1), both with a high *FocSP1* expression level (Fig. 1H). Upon *Foc* TR4 infection, the oeFocSP1 plants exhibited significantly more severe disease symptoms compared with the WT plants (Fig. 1I and J), suggesting that FocSP1 enhances *Foc* TR4 infection. Given the compromised virulence of the *FocSP1* knockout mutant and the enhanced immunity suppression of *FocSP1*-expressing plants, we employed a host-induced gene silencing (HIGS) strategy to disrupt *FocSP1* [5]. Firstly, we tested the efficacy of a double-stranded RNA (*FocSP1*-dsRNA) targeting *FocSP1* by applying it to banana leaves. The treatment effectively suppressed *FocSP1* expression (Fig. 1K) and significantly reduced *Foc* TR4-induced necrosis (Fig. 1L). To test the impact of *in planta* suppression of *FocSP1* expression on *Foc* TR4 infection, *FocSP1*-RNAi transgenic lines were generated using the same silencing sequences*.* Small RNA sequencing confirmed the presence of *FocSP1*-specific siRNAs predominantly ranging from 18 to 25 nt in length, with 20-, 21-, and 22-nt small RNAs being the most abundant (Fig. 1M), indicating the expected functionality of the RNAi construct. Subsequently, the susceptibility of *FocSP1*-RNAi transgenic plants against *Foc* TR4 infection was evaluated. Following inoculation, 100% of WT plants showed severe disease symptoms, while only ~33.3% plants showed severe disease symptoms. The majority of *FocSP1*-RNAi plants exhibited mild or no symptoms (Fig. 1N and O). During the infection process, the relative transcript levels of *FocSP1* in FocSP1-RNAi plants were reduced to <50% of those in the WT controls at both 4 and 8 dpi (Fig. 1P). Collectively, these findings demonstrate that HIGS-mediated silencing of *FocSP1* effectively enhances banana resistance against *Foc* TR4 infections.

To further explore how FocSP1 disrupts the host resistance pathways, we performed transcriptomic analyses on WT, oeFocSP1 (OE) and FocSP1-RNAi (KO) transgenic plants. Compared with WT, substantial gene expression changes were observed in both oeFocSP1 and FocSP1-RNAi transgenic plants, with 1527 differentially expressed genes (DEGs) in oeFocSP1 plants and 888 DEGs in FocSP1-RNAi transgenic plants (Fig. 1Q). Upon *Foc* TR4 infection there were more changes in gene expression: 2926 DEGs were identified between infected FocSP1-RNAi and infected WT plants, while 2431 DEGs were found between infected oeFocSP1 and infected WT plants (Fig. 1Q). Furthermore, DEGs were detected between oeFocSP1 plants infected with *Foc* TR4 and the mock group (yielding 5068 DEGs) or between FocSP1-RNAi plants infected with *Foc* TR4 and the mock group (yielding 4529 DEGs) (Fig. 1Q), suggesting that *FocSP1* expression has a pronounced impact on banana transcriptomes during pathogen challenge. Next, gene ontology (GO) analyses across seven pairwise comparisons (WT_OE, WT_KO, WT_WT^*^, OE_OE^*^, KO_KO^*^, WT^*^_KO^*^, WT^*^_OE^*^) revealed that DEGs were significantly enriched in general plant defense (GO: 0050896, GO: 0009719 and GO: 0006950), phytohormone regulation (GO: 0009725) and ROS regulation (GO: 1901700) (Fig. 1R). Notably, in the WT_KO comparison enriched GO terms were mainly related to phytohormone regulation (GO: 0009723, GO: 0009751, GO: 0009753) and ROS regulation (GO: 0000302, GO: 0006979), and only associated with a few genes. Importantly, this comparison did not show enrichment in general plant defense (Fig. 1R). These findings suggest that FocSP1 modulates host immune responses, particularly by altering the expression of genes involved in plant defense pathways, phytohormone signaling and ROS regulation. The impact of FocSP1 in fungal infection highlights its role as a virulence factor that suppresses plant immunity. To further elucidate the mechanisms, we demonstrated that FocSP1 interacts with an RNA binding protein, MaRBP1, through yeast two-hybrid and luciferase complementation imaging assays (Fig. 1S and T), suggesting that FocSP1 might disturb the host RNA-related processes.

In conclusion, our findings demonstrate that the fungal effector FocSP1 is one of the key virulence factors during *Foc* TR4 infection. FocSP1 contributes to virulence by dysregulating the banana immunity-related genes, thereby facilitating *Foc* TR4 infection. Conversely, HIGS-mediated suppression of FocSP1 expression maintains the functionality of the host immune system and effectively mitigates disease progression by reducing the virulence of *Foc* TR4. Furthermore, the interaction between FocSP1 and the banana RNA-binding protein MaRBP1 highlights the critical role of the FocSP1–MaRBP1 module in manipulating plant immunity and the importance of maintaining a balance in host immune responses for disease resistance. Thus *FocSP1* and *MaRBP1* emerge as promising targets for future resistance breeding programs. Nevertheless, further investigations are required to elucidate how FocSP1 hijacks MaRBP1 to suppress host immunity and which downstream defense pathways are compromised. These findings provide valuable insights into molecular disease-resistance breeding in bananas and may pave the way for developing double-stranded RNA fungicides to control crop fungal diseases.

## Data Availability

The data are accessible via the National Genomics Data Center (NGDC) database at https://ngdc.cncb.ac.cn/ under accession number PRJCA052636.
